# A rare functional cardioprotective *APOC3* variant has risen in frequency in distinct population isolates

**DOI:** 10.1038/ncomms3872

**Published:** 2013-12-17

**Authors:** Ioanna Tachmazidou, George Dedoussis, Lorraine Southam, Aliki-Eleni Farmaki, Graham R. S. Ritchie, Dionysia K. Xifara, Angela Matchan, Konstantinos Hatzikotoulas, Nigel W. Rayner, Yuan Chen, Toni I. Pollin, Jeffrey R. O’Connell, Laura M. Yerges-Armstrong, Chrysoula Kiagiadaki, Kalliope Panoutsopoulou, Jeremy Schwartzentruber, Loukas Moutsianas, UK10K consortium, Emmanouil Tsafantakis, Chris Tyler-Smith, Gil McVean, Yali Xue, Eleftheria Zeggini

**Affiliations:** 1 Wellcome Trust Sanger Institute, Hinxton CB10 1SA, UK; 2 Department of Dietetics-Nutrition, Harokopio, University of Athens, Athens 17671, Greece; 3 Wellcome Trust Centre for Human Genetics, Oxford OX3 7BN, UK; 4 European Molecular Biology Laboratory, European Bioinformatics Institute, Hinxton, CB10 1SD, UK; 5 Department of Statistics, University of Oxford, Oxford OX1 3TG, UK; 6 Division of Endocrinology, Diabetes, and Nutrition, University of Maryland School of Medicine, Baltimore, Maryland 21201-1595, USA; 7 Anogia Medical Centre, Anogia 74051, Greece

## Abstract

Isolated populations can empower the identification of rare variation associated with complex traits through next generation association studies, but the generalizability of such findings remains unknown. Here we genotype 1,267 individuals from a Greek population isolate on the Illumina HumanExome Beadchip, in search of functional coding variants associated with lipids traits. We find genome-wide significant evidence for association between R19X, a functional variant in *APOC3*, with increased high-density lipoprotein and decreased triglycerides levels. Approximately 3.8% of individuals are heterozygous for this cardioprotective variant, which was previously thought to be private to the Amish founder population. R19X is rare (<0.05% frequency) in outbred European populations. The increased frequency of R19X enables discovery of this lipid traits signal at genome-wide significance in a small sample size. This work exemplifies the value of isolated populations in successfully detecting transferable rare variant associations of high medical relevance.

## Abstract (web summary)

Isolated populations may empower genetic association studies of complex traits. Here, the authors identify a rare cardioprotective *APOC3* variant in a Greek population isolate and highlight the value of using population isolates to detect rare variants that confer disease risk.

## Graphical abstract

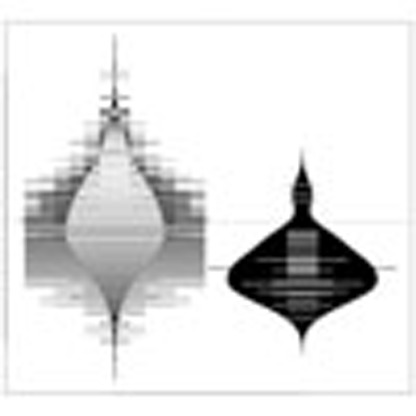

The escalating burden of cardiovascular and metabolic disease has growing health economics and societal implications. Blood lipid levels are measurable indices strongly associated with cardiometabolic disease endpoints. Genetic association studies have in the last few years substantially enhanced our understanding of factors underlying traits of high clinical importance, such as body mass index, lipid levels and blood pressure. Population isolates have been proposed as useful tools in genetic association studies for complex traits, as they are characterized by reduced phenotypic, environmental and genetic heterogeneity^1^. Furthermore, isolated populations can empower next generation association studies focusing on rare variation^2^, as trait-associated alleles may have drifted to higher frequency. However, the generalizability of novel associations identified in population isolates has been the subject of debate. Variants private to the isolate may be highly relevant for that particular population but of limited transferability to other populations. For example, a null *APOC3* variant, R19X, found to be associated with high-density lipoprotein (HDL) and triglycerides levels in the Amish was deemed to be population-specific as it was not detected in non-Amish individuals of European descent given available data at the time^3^.

There is growing empirical evidence that low-frequency and rare variants have an important role in complex human phenotypes. The Illumina HumanExome Beadchip has been developed as a cost-effective tool to study the role of exonic variants and has been successfully applied to the identification of new loci influencing insulin processing and secretion^4^.

To identify potentially functional coding variants associated with cardiometabolic-related traits, we perform exome chip genotyping of 1,267 individuals from a Greek population isolate stemming from Anogia and the surrounding Mylopotamos villages on Crete (HELIC (Hellenic Isolated Cohorts) MANOLIS (Minoan Isolates) study, http://www.helic.org). The Mylopotamos villages’ residents anecdotally demonstrate high rates of longevity. Although Cretans have low mortality rates from cardiovascular disease^5^, their serum lipids are comparable to Northern Europeans^6^,^7^.

We find genome-wide significant evidence for association between R19X, a functional variant in *APOC3*, and increased HDL and decreased triglycerides levels. R19X is rare (<0.05% frequency) in outbred European populations, whereas its frequency has increased to 1.9% in MANOLIS, which enables discovery of this lipid traits signal at genome-wide significance in a small sample size. Our findings additionally address the generalizability of associations discovered in population isolates, as this cardioprotective variant was previously thought to be private to the Amish founder population.

## Results

### Exome chip-wide association analysis

**Figure 1:**
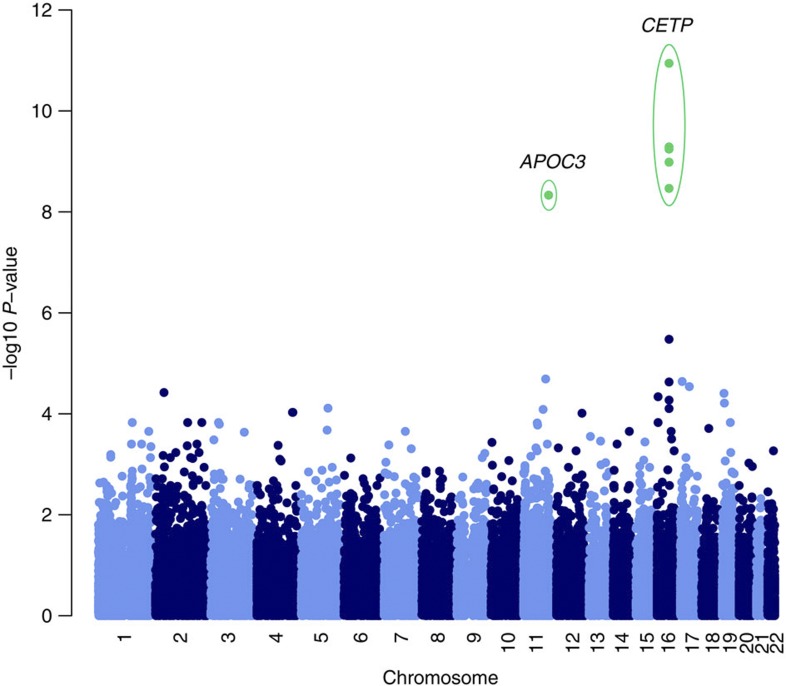
Manhattan plot for HDL in MANOLIS. Genome-wide statistical association evidence for HDL in MANOLIS. *P*-values are generated from the likelihood ratio test, as calculated by GEMMA software.

**Figure 2:**
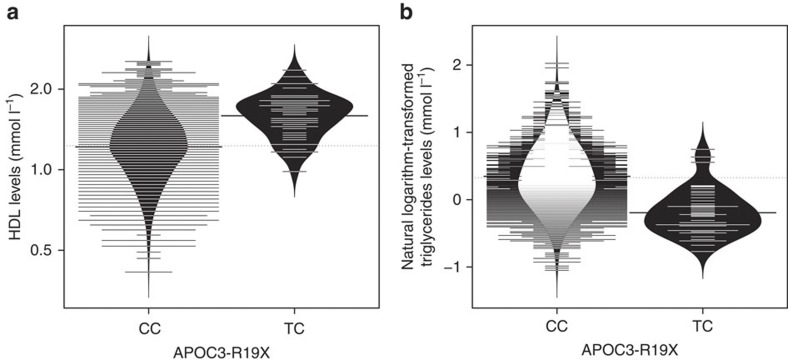
Bean plot of HDL and triglycerides by *APOC3* R19X genotype. The *y*-axis shows the untransformed HDL (**a**) levels (mmol l^−1^) and the natural logarithm-transformed triglycerides (**b**) levels (mmol l^−1^), respectively. HDL *P*=4.65 × 10^−9^ and triglycerides *P*=1.10 × 10^−11^. *P*-values are generated from the likelihood ratio test, as calculated by GEMMA software.

In an analysis of HDL levels using exome chip data in 1,256 individuals from the MANOLIS study (Supplementary Table S1), we find genome-wide significant evidence for association with common-frequency positive control variants in *CETP* (for example, rs1532624, *P*=1.1 × 10^−11^, likelihood ratio test), the well-established HDL-associated locus which has accrued robust evidence for association across multiple populations (Fig. 1; Supplementary Fig. S1; Table 1)^8^,^9^,^10^,^11^,^12^. Despite the small sample size, we also find genome-wide significant evidence for association with a functional variant in *APOC3* (rs76353203, R19X, *P*=4.6 × 10^−9^, likelihood ratio test), which explains 2.9% of the trait variance (Table 2; Fig. 2). The strongest R19X variant association is with decreased triglyceride levels (*P*=1.1 × 10^−11^, likelihood ratio test) and explains 3.9% of trait variance (Table 2; Fig. 2). This clear cardioprotective effect is recapitulated in the strong association with high HDL as a dichotomized trait defined as >60 mg dl^−1^ (ref. 13) (*P*=4.3 × 10^−10^; Table 2, likelihood ratio test). We observe a trend for the minor allele frequency (MAF) to increase with age (Supplementary Fig. S2). There was no evidence for the association of R19X with C-reactive protein (CRP) levels, anthropometric, glycaemic and blood pressure traits and no attenuation of the signal was observed when conditioning on smoking status (Table 2; Supplementary Table S2). The single nucleotide protein (SNP) that led to the original R19X discovery in the Amish^3^ is located in the *DSCAML1* gene (rs10892151) and was highly correlated to R19X in that founder population (*D*′=1, *r*^2^=0.85). In the MANOLIS cohort, we find that the two variants are poorly correlated (*r*^2^=0.001), whereas only three of the possible four haplotypes are observed (*D*′=1, due to low allele frequency).

The T allele, which is the derived allele, changes an arginine residue into a stop codon in exon 2 of all three coding transcripts of the *APOC3* gene. The introduction of a premature stop codon at this position means that the mRNA transcripts are likely to be subject to non-sense-mediated decay and will not be translated.

### R19X frequency and haplotype analysis

R19X has a frequency of 1.9% in the MANOLIS cohort, but is very rare (0.035% frequency; T:3, C:8585) in 4,924 European-descent exomes from the NHLBI data (Exome Variant Server, NHLBI, http://evs.gs.washington.edu/EVS/ (accessed February 2013)) and is not present in the 1,000 Genome Project data^14^. The R19X minor allele is carried by 4 out of 3,621 whole genome sequenced individuals in the UK10K study ( http://www.uk10k.org) (MAF=0.05%). Individuals with the R19X variant do not tend to be closely related in MANOLIS, with 97.3% of carrier pairs having ≤10% of alleles identical by descent exome array-wide (Supplementary Table S3).

The age of the R19X variant in the general European population is estimated at 3.0KYA (95% CI 0–9.0KYA)^15^, which supports the idea that this variant was present at a low frequency in Europe before this Cretan isolate was established. To test this, we examined local haplotypes from UK10K and find that the four chromosomes with the T (stop) alleles share the same ~700 kb haplotype (~200 kb-stop-~500 kb) as we might expect given the frequency of the allele and its age estimated from this frequency. The MANOLIS carriers also all share the same ~400 kb downstream haplotype, which matches ~400 kb of the ~500 kb UK10K haplotype, and some share the entire region. We also examined the Amish T carrier haplotype data and found that the Amish also share almost the same haplotype (181 kb-stop-390 kb) (Supplementary Fig. S3). These findings suggest that R19X has a single recent origin in Europe that predates the establishment of the MANOLIS and Amish isolates, and that it has risen to a relatively high frequency in the two isolates due to genetic drift.

To examine patterns of haplotype sharing around R19X more broadly in the MANOLIS cohort, we identified stretches of maximal haplotype identity along chromosome 11 between pairs of samples using an adaptation of the long-range phasing algorithm of Kong *et al*.^16^ As expected from an isolated population, haplotype sharing is extensive, such that the median size of maximal haplotype identity around a randomly sampled chromosomal location is 14.8 Mb (15.3 cM). However, around R19X, haplotype sharing is no more extensive than elsewhere on the chromosome, nor is it greater among carriers than non-carriers (Kolmogorov–Smirnov test *P*=0.8; Supplementary Fig. S4). Moreover, there is no evidence for cryptic relatedness, substructure or for any extended correlation between genetic background and carrier status (Supplementary Fig. S5), as excess relatedness decays exponentially away from the risk locus as expected. Around R19X, the profile of maximal haplotype identity is well-approximated by a model in which the common ancestor to the genealogical nearest neighbour occurred an average of 4.1 generations ago. This provides a lower bound for the age of the mutation at c. 100 years ago assuming a generation time of 25 years. Interestingly, around other variants of the same frequency, we find greater levels of cryptic relatedness (Supplementary Fig. S6), suggesting that these are typically more recent.

### *APOC3* variant associations with lipid traits

Functional coding mutations in the *APOC3* locus have previously been implicated in several traits related to triglycerides and HDL cholesterol levels^17^,^18^,^19^,^20^,^21^. Variants in the promoter of this gene have also been associated with hypertriglyceridemia^22^,^23^, changes in response to insulin regulation^24^,^25^, and in the case of rs2542052, with low serum APOC3 levels and longevity^26^. We find no evidence for association between rs2542052 and HDL level in the MANOLIS cohort (*P*=0.76, likelihood ratio test).

A large-scale meta-analysis of genome-wide association studies across ~100,000 individuals has also identified robust associations between a common-frequency variant (rs964184) 51.5 kb upstream of *APOC3* and HDL and triglycerides levels^12^. We find little evidence for linkage disequilibrium between rs964184 and R19X (*r*^2^=0.01, *D*′=0.34) in the MANOLIS cohort (Supplementary Table S4), indicating that these are two distinct signals within the same locus. This is further supported by conditional analysis (Supplementary Table S5).

## Discussion

In this study, we examine a newly characterized population isolate from Crete in Greece. Our findings in the Greek isolate demonstrate the utility of the exome array in the discovery of low-frequency variant associations in traits of medical relevance. We find R19X to be associated with increased HDL and reduced triglycerides levels at genome-wide significance. This work provides insights into the allelic architecture of the *APOC3* locus, in which common variants have also been found to be associated with lipid traits by genome-wide association study (for example, Teslovich *et al*.^12^) and highlights the dramatic gains in power that can be afforded by studying population isolates. The R19X functional rare variant has risen in frequency to ~2% in this population, enabling discovery of this lipid traits signal at genome-wide significance in a small sample size. The equivalent sample size needed to achieve 80% power to detect the observed effect size in an outbred European population would be 67,000. Our findings additionally address the generalizability of associations discovered in population isolates both at the gene and at the variant level. The R19X variant has also risen in frequency and shows association with HDL in the Amish founder population^3^. A summary statistic-based meta-analysis across the MANOLIS and Amish^3^ population isolates for R19X leads to a combined *P-*value of 8.1 × 10^−32^, achieved with a total sample size of ~2,700. *APOC3* R19X constitutes to our knowledge the first known example of a clinically important variant that was previously thought to be private to a population but which has in fact drifted in frequency in two independent population isolates and is strongly associated with traits of high clinical relevance. Next generation association studies in search of low frequency and rare variants associated with complex disease can be greatly assisted by the study of population isolates.

## Methods

### Anogia population history

Anogia is a geographically isolated mountainous village on the island of Crete, Greece (population size 4,000). It is located on mount Idi and is part of the municipality of Mylopotamos, which includes further isolated villages. The name Anogia means ‘Upper Earth’ and refers to the altitude at which the village is located (740 m). The residents of Anogia demonstrate characteristic Cretan resolve, great pride in their land and uphold ancient traditions. In 1593, the settlement numbered 911 citizens. Anogia is historically renowned for its residents’ resistance to conquerors. The village has been built three times, as it was burnt down twice by the Turks (in 1822 and 1866) and once by the Germans (in 1944). The local dialect is still strongly influenced by the ancient Dorian language.

### HELIC cohort collection

The HELIC-MANOLIS collection focuses on Anogia and surrounding Mylopotamos villages. Recruitment of this population-based sample was primarily carried out at the village medical centres. All individuals were older than 17 years and had to have at least one parent from the Mylopotamos area. The study includes biological sample collection for DNA extraction and lab-based blood measurements, and interview-based questionnaire filling. The phenotypes collected include anthropometric and biometric measurements, clinical evaluation data, biochemical and haematological profiles, self-reported medical history, demographic, socioeconomic and lifestyle information. Biochemical measurements were obtained using enzymatic colorimetric assays and included glucose (hexokinase method), total cholesterol (cholesterol oxidase – phenol aminophenazone method), HDL-cholesterol, triglycerides (glycerol-3-phosphate oxidase-phenol aminophenazone) and iron. Insulin and ferritin were measured via chemiluminescence and CRP through an immunoturbidimetric method. Low-density lipoprotein (LDL)-cholesterol levels were calculated according to Friedewald equation^27^. The study was approved by the Harokopio University Bioethics Committee, and informed consent was obtained from human subjects.

### Genotyping and QC

Samples were genotyped using the Infinium HumanExome and genotypes were called using Illumina Genome Studio Gencall followed by zCall^28^. We undertook a staged quality control (QC) approach; briefly, we performed an initial prefilter on the GenCalled data excluding samples and variants that had call rate <90%. We carried out a pre-zCall sample QC, excluding samples that had <98% call rate or that were heterozygosity outliers based on the distribution at two different MAF thresholds (MAF <1% and MAF ≥1%). We also excluded samples that were gender discordant and samples that had genotype discordances when we compared these exome chip genotypes to genome-wide genotypes that were established previously using the Illumina OmniExpress platform. No variant exclusions were made at this stage because zCall performs variant QC (which includes call rate >99%, MAF >5%, Hardy Weinberg Equilibrium (HWE) <0.00001). Following zCall, we performed variant QC using the GenCalled genotypes (call rate <95%, HWE *P*<0.0001, cluster separation score <0.4). Sample QC was then carried out on the zCall data excluding samples that were ethnicity outliers or duplicates, that had a call rate <99%, or that were heterozygosity outliers, performed at two different MAF thresholds (MAF <1% and MAF ≥1%). We excluded variants with a zCall call rate <99%. We applied a final sample QC step examining again the multidimensional scaling results, MAF <1% heterozygosity distribution in combination with the number of singletons per sample, and excluded combined visual outliers and any samples that had a minor allele count of 1 for >100 variants. We also applied a final HWE step excluding variants with HWE *P*<0.0001. A total of 1,267 samples and 61,638 variants that were not monomorphic passed QC (Supplementary Table S1).

### Trait transformations and analysis

Trait values were considered to be outliers if they were five standard deviations away from the average value, and were removed from the analysis. Also, LDL values were excluded for individuals with triglycerides level of >400 mg dl^−1^. All phenotypes were analysed directly, apart from triglycerides, insulin, CRP and ferritin, which were normalized using the natural logarithm transformation. Age was inverse normalized. An individual was considered to have high HDL if HDL was over or equal to 60 mg dl^−1^, whereas low HDL was defined as HDL <40 mg dl^−1^. We performed trait association analysis using GEMMA^29^ which incorporates a kinship matrix to account for the relatedness between individuals. *P*-values were produced using the likelihood ratio test. The kinship matrix was generated using exome array genotype data. For HDL and triglycerides, we performed a three-way meta-analysis using the Fisher’s method across the MANOLIS data and the results reported by Pollin *et al*.^3^ (809 Old Order Amish individuals from the Heredity and Phenotype Intervention Heart Study, and 698 Amish individuals from the Amish Family Calcification Study).

### Haplotype analysis to estimate the variant age

Four individuals in the UK10K data set each have one stop allele at R19X. We extracted 1 Mb haplotypes each side of the variant from these individuals as well as from 20 other randomly chosen individuals from the UK data set using only the sites which overlapped with the MANOLIS data set. They shared the same ~700 kb haplotype (~200 kb-stop-~500 kb) as we might expect given the frequency of the allele and its age estimated from this frequency (3.0 KYA, 95% CI 0–9.0 KYA). We then constructed haplotypes for the 48 chromosomes with the stop allele in MANOLIS in the same way. One major inferred ancestral haplotype (accounting for 99/3,740 haplotypes (2.6%) in 99/1,870 individuals (5.3%) of the Amish cohort) with 53 SNPs from Illumina HumanExome Beadchip data was compared with the shared haplotype between UK10K and MANOLIS. Forty-five SNPs were shared among the three data sets. The Amish haplotype shares at least 571 kb (181 kb-stop-390 kb, 116,520,527–117,091,609) with the HELIC and UK10K haplotypes. There are no SNPs in the Amish data between 116,973,929 and 117,100,594 where the HELIC-UK10K shared haplotype ends, and the next SNP available in the Amish data shows that the haplotype is different from the UK10K haplotype. The shared haplotype between the Amish and HELIC-UK10K may extend some way beyond 117,091,609.

### Identification of maximal haplotype sharing

To identify stretches of extended haplotype sharing between individuals, we used a modification of the long-range phasing method of Kong *et al.*^16^ Briefly, for each target individual, we identify a pair of other individuals from the sample as surrogate parents, such that the target individual’s genotype is compatible with Mendelian transmission from the pseudo-parents. We allow for a low level of genotyping error (here 1%) and recombination (here proportional to the fine-scale HapMap genetic map^30^, such that between any pair of SNPs, one of the pseudo-parents can change). We find the most likely parent-pair path using the Viterbi algorithm and reconstruct shared haplotype lengths. Informally, the algorithm can be thought of as attempting to find, for each of an individual’s two chromosomes, those other samples that contain genealogical nearest neighbours. In isolated populations, the method can be used to detect cryptic relatedness and stratification within a sample. The analysis was restricted to those with low levels of relatedness (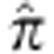
<0.2; 754 individuals of whom 32 are carriers for the risk variant; results are similar with 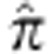
<0.1).

## Author contributions

Cohort collection: G.D., A.-E.F., C.K., E.T. and E.Z. Phenotype cleaning: A.-E.F., K.H., A.M., N.W.R., I.T. and E.Z. Bioinformatics: G.R.S.R., L.S., I.T. and E.Z. Genotype data processing and cleaning: K.H., A.M., K.P., N.W.R., J.S., L.S., I.T. and E.Z. Genotype–phenotype association testing: L.S., I.T. and E.Z. UK10K haplotype analyses: Y.C., UK10K Consortium, Chris Tyler-Smith, Yali Xue. Amish haplotype analysis: T.I.P., J.R.O’C., L.M.Y.-A. and Y.X. HELIC haplotype sharing: G.M., L.M. and D.K.X. Manuscript drafting: G.R.S.R., L.S., I.T., D.K.X., Y.X. and E.Z.

## Additional information

**How to cite this article:** Tachmazidou, I. *et al.* A rare functional cardioprotective *APOC3* variant has risen in frequency in distinct population isolates. *Nat. Commun.* 4:2872 doi: 10.1038/ncomms3872 (2013).

**Accession codes:** The HELIC-MANOLIS study genotype data have been deposited to the European Genome-phenome Archive (EGA) under the accession code EGAS00001000630. UK10K sequence data have been deposited to the EGA under the accession codes EGAS00001000108 and EGAS00001000090.

## Supplementary Material

Supplementary Information Supplementary Figures S1-S6, Supplementary Tables S1-S5 and Supplementary Note 1

## Figures and Tables

**Table 1:** *CETP* variants associated with HDL levels in MANOLIS.

**rs ID**	**Chromosome**	**Position**	**Effect allele**	**Effect allele frequency**	**Beta**	s.e.*	***P*****-value**†
rs173539	16	56988044	T	0.2924	0.1007269	0.01689119	3.46 × 10^−9^
rs247616	16	56989590	T	0.2833	0.1044224	0.01694992	1.03 × 10^−9^
rs3764261	16	56993324	A	0.2861	0.1064031	0.01695299	5.16 × 10^−10^
rs1800775	16	56995236	A	0.4514	0.09163417	0.01461291	5.66 × 10^−10^
rs1532624	16	57005479	A	0.4163	0.103284	0.01503399	1.13 × 10^−11^

^*^Standard error.

^†^*P*-values are calculated using the likelihood ration test, as calculated by the GEMMA software.

**Table 2:** Distribution of lipid traits by R19X genotype.

**Trait**	CC*	CT*	**Effect size**†	***P*****-value**‡
*N*	1,219	48	—	—
Sex (M:F)	518:699	23:24	0.943 (0.813, 1.093)	0.435
Age (years)§	62.058 (19.51)	66.234 (17.20)	5.257 (2.944)	0.189
HDL (mmol l^−1^)	1.265 (0.35)	1.619 (0.30)	0.319 (0.054)	4.65 × 10^−9^
LDL (mmol l^−1^)	3.272 (0.94)	3.205 (0.73)	−0.042 (0.143)	0.767
Total cholesterol (mmol l^−1^)	5.288 (1.10)	5.225 (0.79)	−0.047 (0.167)	0.778
Triglycerides (mmol l^−1^)	0.313 (0.49)	−0.220 (0.33)	−0.513 (0.075)	1.10 × 10^−11^
High HDL	232/1209 (0.192%)	28/47 (0.596%)	1.471 (1.305,1.659)	4.29 × 10^−10^
Low HDL	307/1209 (0.254%)	1/47 (0.021%)	0.812 (0.713,0.924)	1.64 × 10^−3^

^*^Untransformed variables are presented as mean (s.d.) and natural logarithm-transformed variables are presented as median (s.d.).

^†^For continuous traits beta values (standard error) are reported, whereas for binary traits odds ratios and 95% confidence intervals are reported.

^‡^*P*-values are calculated using the likelihood ratio test, as calculated by the GEMMA software.

^§^Inverse-normalized for analysis.
