# Supplementary material for: A rare functional cardioprotective APOC3 variant has risen in frequency in distinct population isolates
Source: Nat Commun. 2013 Dec 17;4:2872. doi: 10.1038/ncomms3872 (PMC3905724; doi:10.1038/ncomms3872)
Supplement: Supplementary Information — Supplementary Figures S1-S6, Supplementary Tables S1-S5 and Supplementary Note 1 [file ncomms3872-s1.pdf]

Supplementary Figures

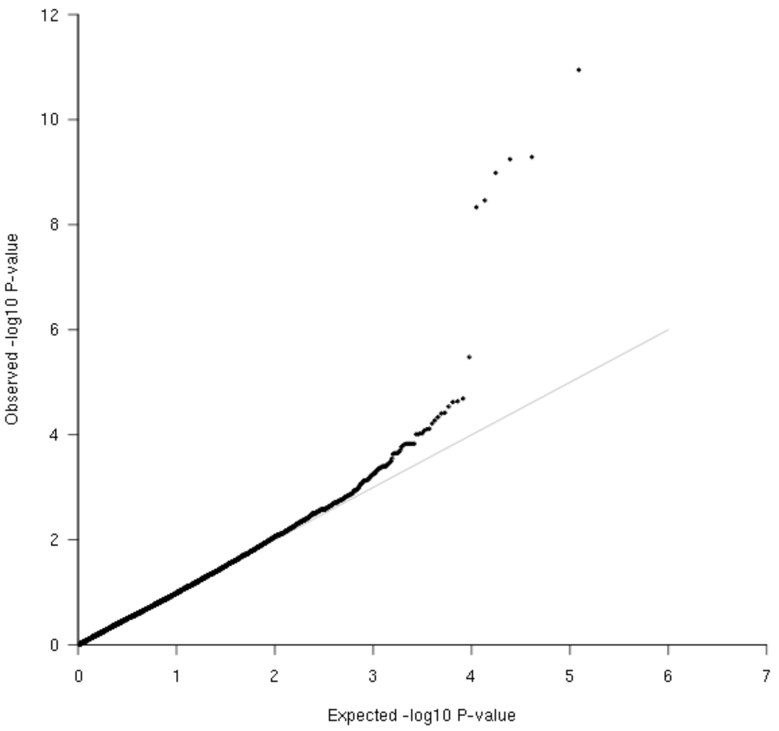

Supplementary Figure S1. Q-Q plot for HDL levels in MANOLIS. P-values are generated from the likelihood ratio test, as calculated by GEMMA software.

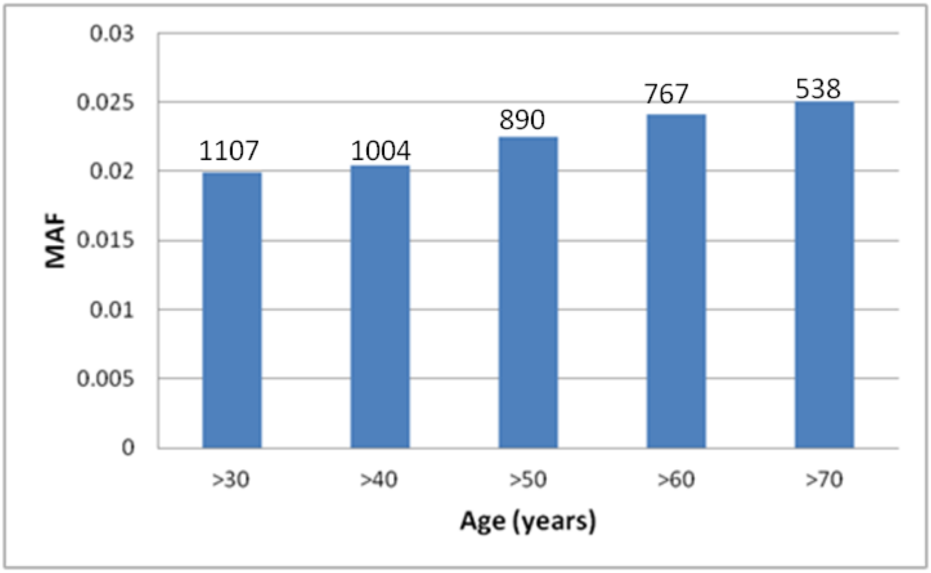

Supplementary Figure S2. Histogram of minor allele frequency for R19X by age group in MANOLIS. Minor allele frequency tends to increase for R19X with age.

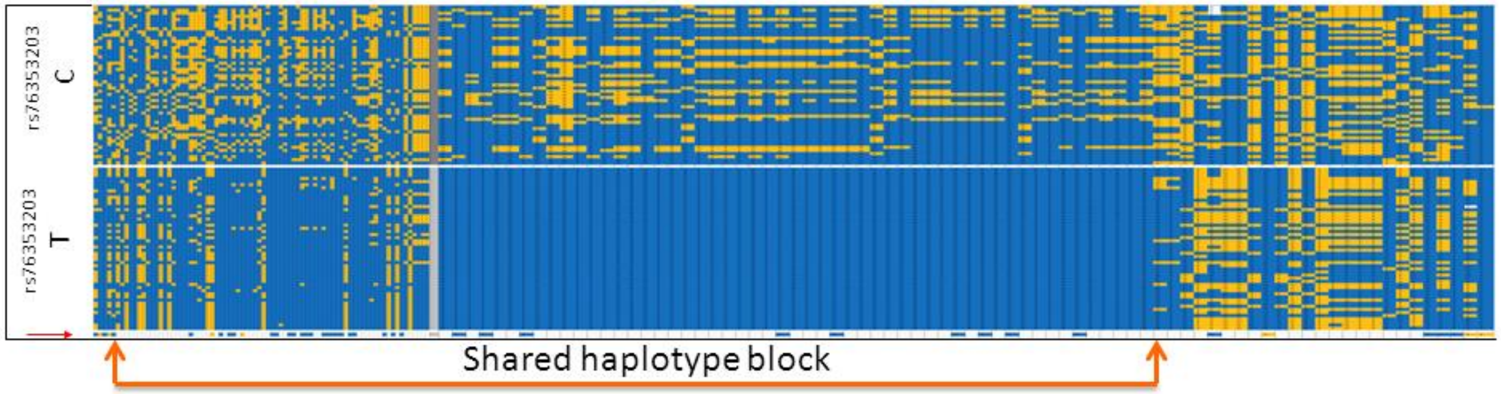

Supplementary Figure S3. Haplotype structure in the individuals carrying the T (stop) allele at *APOC3* R19X (rs76353203) inferred from 157 common SNPs. Each row represents one chromosome; haplotypes are split into two panels: rs76353203 C and rs76353203 T. The top 48 rows of each panel represent individuals from the HELIC-MANOLIS cohort, and the bottom 4 rows represent UK10K individuals. The final row, indicated by the red arrow on the left, is the inferred haplotype of the chromosomes with the rs76353203 T variant in the Amish cohort; 45 out of 53 SNPs typed in the Amish overlapped with the HELIC-MANOLIS cohort and UK10K. Each column represents a variant, with the major allele (calculated in these 52 samples) in blue and the minor allele in orange. The missing positions in the Amish samples are shown in white. *APOC3* R19X (rs76353203) is in grey.

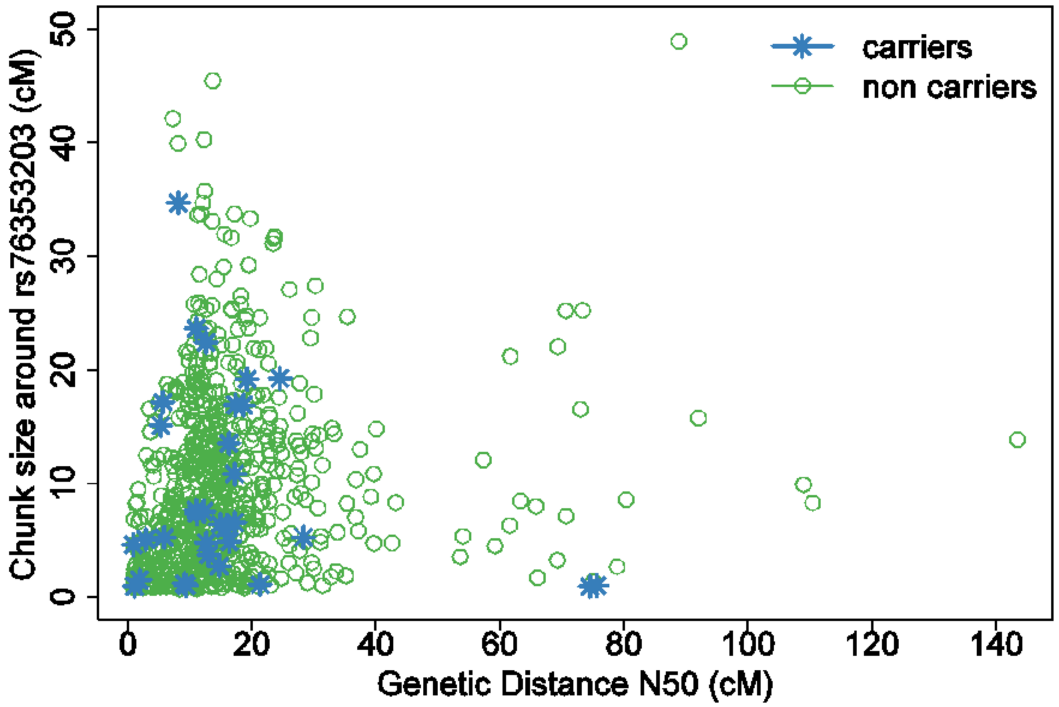

Supplementary Figure S4. Scatterplot showing the relationship between the median maximal haplotype sharing for each sample across the chromosome and at the risk variant. X-axis is referred to as the N50 haplotype length as it is equal to the length of shared segment for which 50% of all positions along the chromosome lie in. Carriers (blue) and non-carriers (green) are shown separately. Note that carriers and non-carriers show similar patterns in both statistics (no significant difference, p>0.05, by Kolmogorov-Smirnov test).

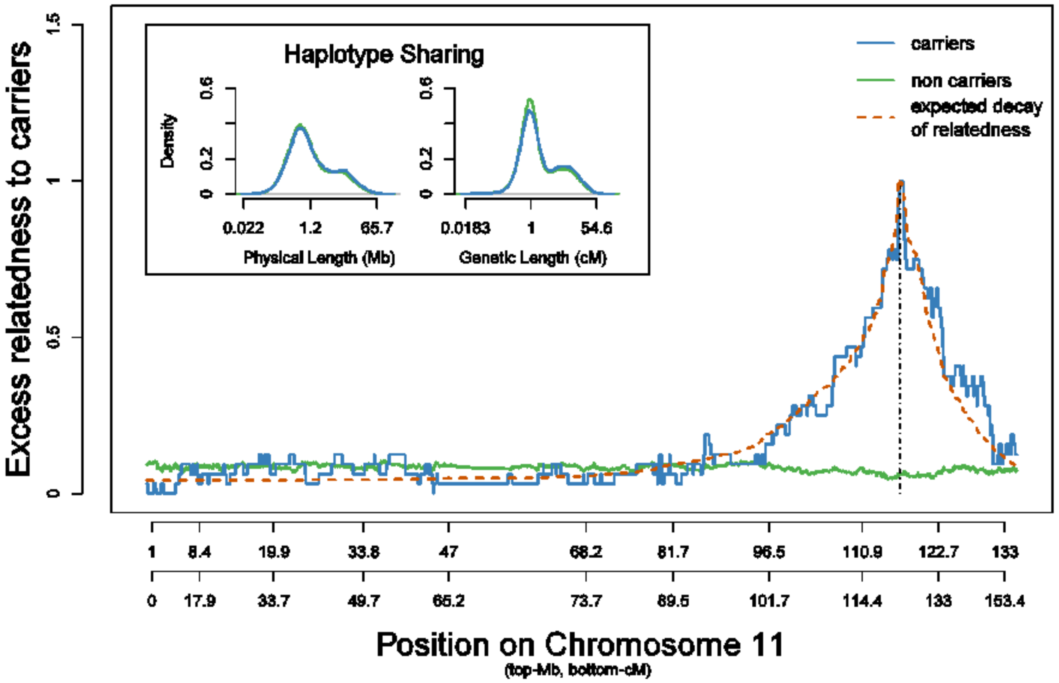

Supplementary Figure S5. Maximal haplotype sharing around *APOC3*. The main plot shows the average number of nearest neighbors that are carriers of the rare risk allele for carriers (blue) and non-carriers (green). Analysis only for unrelated samples (pi-hat<0.2, see Methods). The red line shows the expectation from a model when all most recent common ancestor events to the nearest neighbor occurred 4.1 generations ago. The inset shows the distributions of maximal shared haplotype length (on a log scale) in physical (left) and genetic (right) distance for carriers (blue) and non-carriers (green).

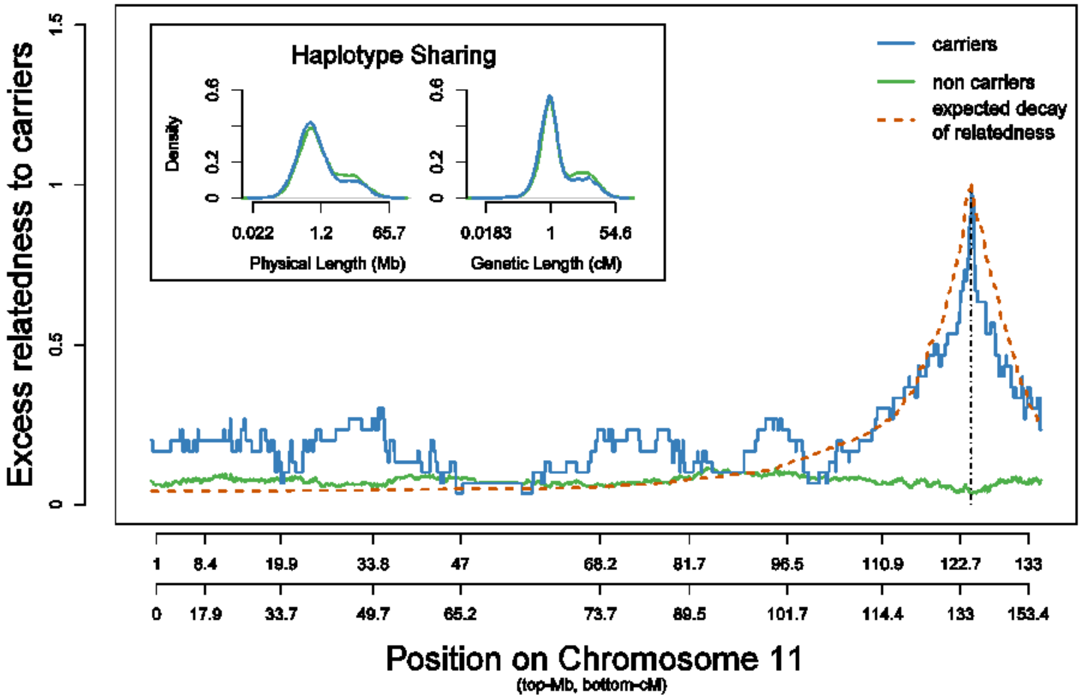

Supplementary Figure S6. Maximal haplotype sharing around other variants on chromosome 11 where the derived allele is at a similar frequency as the risk allele in *APOC3*. In each case, the main plot shows the average number of nearest neighbors that are carriers of the rare risk allele for carriers (blue) and non-carriers (green). The red line shows the expectation from a model when all most recent common ancestor events to the nearest neighbor occurred 4.1 generations ago. The inset shows the distributions of maximal shared haplotype length (on a log scale) in physical (left) and genetic (right) distance for carriers (blue) and non-carriers (green).

Supplementary Tables

Supplementary Table S1. Minor allele frequency distribution of exome chip variants passing quality control in the MANOLIS cohort (n=1267).

MAF category	n	%[a]
>0 - <0.005	20,596	33.41
≥0.005 - <0.01	5324	8.64
≥0.01 - <0.05	9717	15.76
≥0.05 - <0.1	4125	6.69
≥0.1 - <0.2	5607	9.1
≥0.2 - <0.3	4873	7.91
≥0.3 - <0.4	5251	8.52
≥0.4 - ≤0.5	6145	9.97
All excl. monomorphic	61,638	
Monomorphic	172,935	73.72
All incl. monomorphic	234,573	

[a]The proportion of variants in different MAF categories is calculated based on the total number of non-monomorphic variants passing quality control. The proportion of monomorphic variants is calculated based on the total number of variants passing quality control.

Supplementary Table S2. Distribution of anthropometric, glycaemic, blood pressure and other medically-relevant traits by R19X genotype.

Trait	CC[a]	CT[a]	Effect size[b]	p value[c]
Insulin (pmol/l)	4.135 (0.77)	4.423 (0.67)	0.044 (0.122)	0.722
Fasting Insulin (pmol/l)	4.135 (0.70)	4.423 (0.66)	0.122 (0.136)	0.369
Glucose (mmol/l)	5.700 (1.61)	5.643 (1.38)	-0.003 (0.246)	0.993
Fasting Glucose (mmol/l)	5.670 (1.51)	5.426 (0.92)	-0.208 (0.278)	0.453
BMI (kg/m^2)	29.419 (5.16)	30.782 (5.32)	1.148 (0.843)	0.174
Weight (kg)	77.603 (15.50)	80.491 (14.32)	2.784 (2.488)	0.263
Height (cm)	162.436 (9.92)	161.965 (11.22)	-0.048 (1.612)	0.977
WHRf	0.876 (0.09)	0.919 (0.07)	0.031 (0.021)	0.133
WHRm	0.965 (0.08)	0.98 (0.08)	0.015 (0.018)	0.383
WHR	0.914 (0.10)	0.948 (0.08)	0.030 (0.016)	0.054
SBP (mmHg)	138.9 (20.23)	140.762 (23.10)	0.912 (3.304)	0.783
DBP (mmHg)	78.883 (11.17)	76.81 (10.29)	-2.071 (1.802)	0.250
PP (mmHg)	60.017 (17.9)	63.952 (20.05)	2.964 (2.923)	0.312
CRP (mg/l)	0.642 (1.02)	1.03 (1.06)	0.126 (0.158)	0.427
Fe Iron (µg/dl)	85.601 (32.5)	95.404 (37.71)	9.624 (5.028)	0.0565
Ferritin (ng/ml)	4.394 (1.03)	4.533 (0.91)	0.152 (0.157)	0.332
HDL (mmol/l) conditional on current smoking status	1.266 (0.35)	1.631 (0.30)	0.317 (0.054)	$5.35\text{x}10^{-9}$
HDL (mmol/l) conditional on ever smoked status	1.266 (0.35)	1.631 (0.30)	0.318 (0.054)	$4.86\text{x}10^{-9}$
Triglycerides (mmol/l) conditional on current smoking status	0.321 (0.49)	-0.227 (0.33)	-0.510 (0.075)	$1.40\text{x}10^{-11}$
Triglycerides (mmol/l) conditional on ever smoked status	0.321 (0.49)	-0.227 (0.33)	-0.510 (0.075)	$1.34\text{x}10^{-11}$

BMI: body mass index; WHRf: waist hip ratio in females; WHRm: waist-hip ratio in males; SBP: systolic blood pressure; DBP: diastolic blood pressure; PP: pulse pressure; CRP: C-reactive protein. [a]Untransformed variables are presented as mean (standard deviation (sd)) and natural logarithm-transformed variables are presented as median (sd). [b]For continuous traits beta values (standard error) are reported.[c]P-values are calculated using the likelihood ratio test, as calculated by the GEMMA software.

Supplementary Table S3. Distribution of pi-hat (measure of the proportion of alleles IBD exome array-wide) for pairs of R19X carriers.

Threshold	Proportion of pairs with pi-hat>threshold	n unique individuals	Proportion of pairs with pi-hat<=threshold	n unique individuals
0.03125	0.145	44	0.855	3
0.05	0.065	33	0.935	14
0.10	0.027	22	0.973	25
0.125	0.022	21	0.978	26
0.15	0.019	21	0.981	26
0.20	0.016	20	0.984	27
0.25	0.016	20	0.984	27
0.30	0.014	20	0.986	27
0.35	0.013	20	0.987	27
0.40	0.013	20	0.987	27
0.45	0.013	20	0.987	27
0.50	0.006	11	0.994	36
0.55	0.003	6	0.997	41
0.60	0.001	2	0.999	45
0.65	0	0	1	47

Supplementary Table S4. Haplotype frequencies for rs964184 and rs76353203 in the MANOLIS samples. TheR19X cardioprotective allele is denoted in bold.

Haplotype	Frequency
G**T**	0.009
C**T**	0.010
GC	0.168
CC	0.813

Supplementary Table S5. Conditional analysis for rs964184 and R19X.

variant	analysis	HDL p value	Triglycerides p value
rs76353203	unconditioned	$4.65x10^{-9}$	$1.10x10^{-11}$
rs76353203	conditional on rs964184	$1.06x10^{-9}$	$4.80x10^{-13}$
rs964184	unconditioned	$2.85x10^{-2}$	$1.17x10^{-4}$
rs964184	conditional on rs76353203	$5.61x10^{-3}$	$4.60x10^{-6}$

P-values are calculated using the likelihood ratio test, as calculated by the GEMMA software.

Supplementary Note 1: UK10K cohort author list

Inês Barroso[1], Chris Boustred[2], Celia Greenwood[3,4], Lu Chen[1], Gail Clement[5], Petr Danecek[1], George Davey Smith[2], Aaron Day-Williams[1,6], Ian Day[2], Richard Durbin[1], David Evans[2], Ghazaleh Fatemifar[2], Tom Gaunt[2], Audrey Hendricks[1], Jie Huang[1], Yalda Jamshidi[7], David Jewell[2], Chris Joyce[1], John Kemp[2], Genevieve Lachance[5], Rui Li[3,8], Yingrui Li[9], Margarida Lopes[1,10], Massimo Mangino[5], Shane McCarthy[1], Yasin Memari[1], Sarah Metrustry[5], Josine Min[2], Alireza Moayyeri[5], Dawn Muddyman[1], Kate Northstone[2], Kalliope Panoutsopoulou[1], Lavinia Paternoster[2], John Perry[5,10,11,12], Lydia Quaye[5], Brent Richards[3,5,8], Sue Ring[2], Graham Ritchie[1,13], So-Youn Shin[1], Kerrin Small[5], María Soler Artigas[14], Nicole Soranzo[1], Tim Spector[5], Lorraine Southam[1], Beate St Pourcain[2], Jim Stalker[1], Gabriela Surdulescu[5], Ioanna Tachmazidou[1], Jing Tian[9], Nic Timpson[2], Martin Tobin[14], Louise Wain[14], Klaudia Walter[1], Jun Wang[9], Kirsten Ward[5], Scott G. Wilson[5,15,16], Kim Wong[1], Eleftheria Zeggini[1], Feng Zhang[5], Hou-Feng Zheng[3,8]

[1]The Wellcome Trust Sanger Institute, Wellcome Trust Genome Campus, Hinxton CB10 1HH, Cambridge, UK.

[2]MRC CAiTE Centre, School of Social and Community Medicine, University of Bristol, Oakfield House, Oakfield Grove, Clifton, Bristol, BS8 2BN, UK.

[3]Departments of Epidemiology, Biostatistics and Occupational Health, Lady Davis Institute, Jewish General Hospital, McGill University, Montreal, Quebec H3T 1E2, Canada.

[4]Department of Oncology, McGill University, Montreal, Quebec H2W 1S6, Canada

[5]The Department of Twin Research & Genetic Epidemiology, King's College London, St Thomas' Campus, Lambeth Palace Road, London, SE1 7EH, UK.

[6]Department of Translational Sciences, Biogen Idec, 14 Cambridge Center, Cambridge, MA 02142, USA.

[7]Human Genetics Research Centre, St George's University of London, SW17 0RE, UK.

[8]Departments of Medicine & Human Genetics, Lady Davis Institute, Jewish General Hospital, McGill University, Montreal, Quebec H3T 1E2, Canada.

[9]BGI-Shenzhen, Shenzhen 518083, China.

[10]The Wellcome Trust Centre for Human Genetics, Roosevelt Drive, Oxford, OX3 7BN, UK.

[11]Genetics of Complex Traits, Peninsula Medical School, University of Exeter, Exeter EX4 4SB, UK.

[12]Center for Statistical Genetics, University of Michigan, Ann Arbor, Michigan, MI 48109-2029, USA.

[13]European Molecular Biology Laboratory, European Bioinformatics Institute, Wellcome Trust Genome Campus, Hinxton, Cambridge, CB10 1SD, UK.

[14]Departments of Health Sciences and Genetics, University of Leicester, Leicester LE1 7RH, UK.

[15]School of Medicine and Pharmacology, University of Western Australia, Perth, WA 6009, Australia.

[16]Department of Endocrinology and Diabetes, Sir Charles Gairdner Hospital, Nedlands, WA 6009, Australia.
